# The impact of data driven motion correction for clinical brain PET/MRI radiotracers

**DOI:** 10.1186/s40658-026-00863-7

**Published:** 2026-04-15

**Authors:** Martin Bolin, Anna Falk Delgado, Emilia Palmér

**Affiliations:** 1 Centre for Psychiatry Research, Department of Clinical Neuroscience, Karolinska Institutet & Stockholm Health Care Services, Stockholm, Region Stockholm Sweden; 2 Department of Clinical Neuroscience, Karolinska Institutet and Department of Neuroradiology, Karolinska University Hospital, Stockholm, Sweden; 3 Department of Molecular Medicine and Surgery, Karolinska Institutet and Department of Nuclear Medicine and Medical Radiation Physics, Karolinska University Hospital, Stockholm, Sweden

**Keywords:** Motion correction, Data driven motion correction, lmDuetto, Duetto, PET, PET/MR

## Abstract

**Purpose:**

The Positron Emission Tomography (PET)/Magnetic Resonance Imaging (MRI) scanner combines two diagnostic imaging modalities, providing information on anatomy and physiology. Beneficial diagnosis areas are epilepsy ([$^{18}$F]Fluorodeoxyglucose (FDG)) and cancer recurrence ([$^{11}$C]methionine (MET)), where subject motion during PET acquisition reduces image quality, potentially compromising diagnostic accuracy. This project aimed to evaluate the impact of PET data-driven motion correction (ddMC) of these clinical PET radiotracers and to assess, for the first time, whether the automatic motion categorization reflects motion levels impacting the image quality.

**Methods:**

Eighty-nine PET scans (66 [$^{11}$C]MET, 23 [$^{18}$F]FDG) were reconstructed with ddMC and without motion correction (noMC) using the research software lmDuetto toolbox (GE Healthcare, Chicago, IL, USA), and were automatically categorized into motion groups. MRI images were segmented, and the regions of interest (ROIs) transferred to the PET space. The effect of ddMC was analyzed by relative signal differences between ddMC and noMC. Motion estimation and categorization were evaluated by normalized cross correlation (XC) over time and the proposed cumulative displacement-time histogram (cDTH).

**Results:**

Overall, ddMC increased signal values within cortical ROIs compared to noMC. In the high motion category, median relative mean signal differences were 0.61% (0.41−0.80%) for [$^{18}$F]FDG and 0.70% (0.61−0.79%) for [$^{11}$C]MET. The XC improved ([$^{18}$F]FDG: 0.80 to 0.97, [$^{11}$C]MET: 0.85 to 0.98). Low and medium motion groups had lesser impact, indicating motion correction is most relevant for high motion. The XC and cDTH identified subjects whose motion classification should be revised.

**Conclusion:**

In conclusion, the results confirm previous findings with ddMC using [$^{18}$F]FDG and demonstrate its suitability for lower-accumulating [$^{11}$C]MET. The automatic motion categorization needs re-evaluation to better reflect motion affecting PET image quality.

## Introduction

Positron Emission Tomography (PET) is a valuable imaging modality for assessing metabolic and physiological processes. However, PET images often suffer from limited spatial resolution and reduced anatomical detail due to the intrinsic properties of the imaging technique. To address these limitations, the integration of PET and Magnetic Resonance Imaging (MRI) scanners has enabled the simultaneous acquisition of metabolic and high-resolution anatomical information. The use of PET/MR scanners is particularly valuable in diagnosing conditions such as epilepsy or cancer recurrence, where near-perfect alignment of metabolic and anatomical data is crucial due to the presence of relatively small and localized abnormalities. Therefore, the resolution and contrast of the generated images are of great importance. PET images typically exhibit lower resolution (4-6 mm) in comparison to MRI data (1-2 mm). Even at this resolution, minor patient movements of a few millimeters during PET data acquisition can introduce intra-frame motion blurring, compromising the accuracy of the final image.

Several methods for head motion management have been proposed [1–8]. One approach is post reconstruction image-based registration between reconstructed PET frames [3, 9], not requiring any external measurement of the patient motion. Within a PET frame, it is assumed that the tracer distribution is static and the subject is motionless, restricting the effectiveness of the motion correction technique if longer frames (i.e. minutes) are reconstructed. To improve effectiveness, motion estimation approaches, such as the Centroid-of-Distribution (COD) [7], can be used to verify low-motion data segments during image reconstruction. These data segments can then be registered to one another. A more sensitive method for monitoring subject motion is the use of external or physical motion trackers [1, 4–6, 10]. When list-mode reconstruction is applied, the estimated motion from the external motion trackers can be incorporated to correct displacement on an event-by-event basis, thereby enhancing the contrast of the PET image [11–13]. However, physical motion tracking requires the attachment of external markers to the subject, introducing additional steps in the clinical data acquisition process [5, 10]. The marker-less approach eliminates the need for physical markers but requires a dedicated prospective hardware setup and clear camera visibility of the subject, which complicate integration into standard clinical PET/MRI workflows [14]. An alternative approach that avoids external motion trackers and additional steps into the data acquisition process is to estimate motion using a data-driven method [15–17].

A data-driven list-mode motion correction method has been proposed for [$^{18}$F]fluorodeoxyglucose (FDG) brain PET (a pre-version of the commercially available MotionFree Brain; GE Healthcare, Chicago, IL, USA) [15, 18, 19]. In this approach, available through the Duetto research toolbox (GE Healthcare, Chicago, IL, USA) with the list-mode package (lmDuetto), short PET frames (i.e. seconds) are reconstructed and rigidly registered to a common frame of reference, creating motion dependent rigid transformation parameters over time that can be used to estimate rotational and translational motion of each short frame. The motion estimation is subsequently incorporated into the full list-mode PET reconstruction, allowing offline correction of the PET data during the image reconstruction step.

Validation of motion correction for each radiotracer is essential, particularly for radiotracers with low accumulation or challenging distribution patterns. Previous studies have assessed motion correction effects utilizing lmDuetto with a [$^{18}$F]-filled phantom [20], high-accumulating [$^{18}$F]FDG [19], and low-accumulating [$^{18}$F]-MK6240 [21]. Data on the low-accumulating [$^{11}$C]methionine (MET) radiotracer in pediatric brain tumor patients have only recently been reported [14], focusing primarily on global PET metrics within brain lesion. Since region-specific effects of motion correction remain unexplored and brain motion is not purely translational, with a rotation center typically located posteriorly, frontal cortical regions are hypothesized to be more affected than others. This study aims to assess the robustness of data driven list-mode motion correction utilizing lmDuetto for the [$^{11}$C]MET tracer distribution across multiple brain regions and compare it with [$^{18}$F]FDG. Additionally, this work investigates, for the first time for any tracer, whether the commercially proposed automatic motion categorization provided by the lmDuetto software accurately reflects motion levels impacting the PET image quality.

## Materials and methods

### Study cohort

The study was approved by the Swedish Ethical Review Authority (Dnr 2022-01561-01). All subjects received oral and written information prior to inclusion and gave a written consent. Inclusion criteria were subject referred for PET/MR examination of the brain older then 18 years old. Exclusion criteria were inability to consent, aborted exam within the PET acquisition, and corrupt MR images.

### PET imaging and reconstruction

Simultaneous PET and MRI were acquired using a Signa 3T PET/MR scanner (GE Healthcare, Chicago, IL, USA) with a 19-channel head and neck coil. The data used in this study was 15 min (except one dataset of 25 min) list mode PET data acquired 15 or 60 min post injection (clinical routine for [$^{11}$C]MET and [$^{18}$F]FDG, respectively), MRI attenuation correction pulse sequences (Dixon and zero echo time (ZTE), with ZTE used for attenuation correction in all cases), and axial pre- or post-contrast injection T1-weighted 3D ultra fast gradient echo MRI pulse sequence.

The PET list data was reconstructed utilizing the offline reconstruction software lmDuetto Toolbox (v02.18, GE Healthcare, Chicago, IL, USA) for MATLAB (2021b, Mathworks Inc., Natick, MA, USA). For PET motion correction evaluation, the PET data were statically reconstructed four times using two reconstruction algorithms, Ordered Subsets Expectation Maximization with Point Spread Function correction (OSEM-PSF) and Block Sequential Regularized Expectation Maximization (BSREM) (for registration robustness purposes), both with and without motion correction (referred to as ddMC and noMC, respectively). All reconstructions were corrected for attenuation, scatter (using single scatter simulation), randoms, and decay. For the motion estimation, PET data was reconstructed without attenuation and scatter correction, according to the methodology developed by Sprangler-Bickell et al.[15]. During the motion estimation, the acquisition time of the Dixon attenuation correction pulse sequence was used as the reference frame and the short PET frames had constant 5e5 events per frame (e.g. different time lengths, range: 0.9–30 s). For PET motion estimation evaluation, a subset of subject was dynamically reconstructed by 30-seconds OSEM-PSF PET frames, with and without ddMC.

### Image post processing

Image post processing was conducted using an in-house developed evaluation pipeline previously developed by integrating a combination of software packages. The workflow consisted of three main successive nodes: brain segmentation utilizing T1-weighted MRI data (or contrast enhanced T1-weighted MRI data if pre-contrast imaging was unavailable) (Freesurfer, version 6.0, [22]), rigid registration of the MRI data to both ddMC and noMC (SPM12, [23]), and translation of the segmentation to the corresponding PET data (SPM12, [23]).

The rigid registration node was twofold. First, the MRI data was rigidly registered to the ddMC and noMC PET reconstructions using the BSREM algorithm. The accuracy of this registration was visually assessed. Second, for the subsequent MRI-to-PET registration (OSEM-PSF), the transformation matrix from the BSREM registration was used as an initial alignment to improve the robustness of the OSEM-PSF registration. This was followed by evaluating the difference between the BSREM displacement vector of a point source in space and the displacement vectors from the second reconstruction. A MRI-to-PET registration with OSEM-PSF was considered to be successful if the difference was $\le$ 1 mm; otherwise, its accuracy was visually assessed.

### PET motion evaluation

The hypothesis was that ddMC performance would be similar across [$^{11}$C]MET and [$^{18}$F]FDG tracers, that ddMC would have a greater effect in frontal cortical regions than others, that the effect of ddMC varied with motion magnitude, and that automatic motion categorization would reflect motion levels impacting PET image quality.

The subjects were automatically categorized into three groups based on the estimated motion calculated by lmDuetto [19]: low motion (median absolute displacement <1 mm), medium motion (1 mm$\ge$ median absolute displacement $\le$2 mm), and high motion (median absolute displacement >2 mm). The displacement was calculated by defining two points in image space at 70 mm anterior and 70 mm posterior to the brain center, which were moved according to the estimated motion parameters [19]. The median absolute displacement from the reference frame was then computed for each point, with the larger of the two values used for motion categorization.

To evaluate the effect of ddMC across radiotracers, brain regions, and motion magnitudes, the mean signal values within chosen segmented regions of interest (ROIs) were compared between ddMC and noMC OSEM-PSF PET by calculating the relative signal differences ((ddMC - noMC)/ddMC x 100%). Statistical comparisons between radiotracers were conducted using Mann–Whitney U tests to assess whether ddMC performance differed between [$^{11}$C]MET and [$^{18}$F]FDG. A significance level of $\alpha = 0.05$ was set, and Bonferroni correction was applied to adjust for multiple comparisons.

The outcome of the motion estimation and the automatic motion categorization provided by the lmDuetto software was evaluated using the 30-seconds dynamic OSEM-PSF PET frames, both with and without motion correction, followed by calculating the 3D normalized cross-correlation (XC) over time (modified MATLAB code ([4])). Two subjects from each motion category and radiotracer were evaluated, with low and medium groups randomly selected, resulting in a total of 12 subjects. The XC reference frame was selected as a 30-second subset of the PET data temporally matching the ddMC reference time frame. The motion reference frame was visually inspected to ensure no motion was present within the reference. A brain mask was generated by thresholding (30% of signal maximum) the PET data within the XC reference frame after Gaussian filtering (16 mm). This followed the calculation of XC of the noMC and ddMC 30 s PET frames using a 3 mm Gaussian filter. Additionally, to visualize and more easily analyze the motion estimation, cumulative displacement-time histogram (cDTH) was conducted for the 12 subjects, illustrating the proportion of the scan time spent at different displacement magnitudes.

## Results

### Study cohort

By June 2024, a total number of 94 subjects were consecutively enrolled in the study. However, eight of these subjects were later excluded due to the absence of list mode PET data, one subject was excluded due to a different radiotracer, one subject was excluded due to offline reconstruction errors, and one subject encountered segmentation errors due to abnormal anatomy. As a result, 83 subjects (age: 18 – 81 years, sex: 45 male, 38 female, weight: 45 – 128 kg, length: 152 – 192 cm) were eligible for ddMC evaluation. Six subjects underwent repeated PET scans. In total, 89 PET scans were evaluated with 66 [$^{11}$C]MET administrations (prescribed activity: 134-435 MBq) and 23 [$^{18}$F]FDG administrations (prescribed activity: 150-321 MBq).

Out of the 89 PET scans, 66 scans (74%) were categorized as low motion, with 53 of these being [$^{11}$C]MET scans; 19 scans (21%) were classified as medium motion, with eleven being [$^{11}$C]MET; and four scans (5%) were categorized as high motion, with two being [$^{11}$C]MET.

### Image processing

The included subjects were successfully post processed utilizing the in-house developed software. For two subjects, the segmentation was conducted utilizing contrast enhanced T1-weighted MRI data. The accuracy of the rigid registration of MRI to ddMC and noMC was visually estimated to be successful for BSREM. A difference $\le$1 mm between the BSREM displacement vector and the OSEM-PSF reconstruction algorithm was observed for 100% of the registrations, hence all registrations were considered to be successful.

### PET motion evaluation

Evaluating the hypothesis that ddMC would have a greater effect in frontal cortical regions than others, Table 1 presents the evaluated overall and regional effect of ddMC across all subjects and each radiotracer, assessed by calculating the median relative signal difference for each ROI. A positive median relative signal differences indicate that motion correction yields a high signal value within the ROIs. The implementation of motion correction slightly increased signal values within cortical ROIs, while subcortical ROIs showed negligible changes.

**Table 1:** The median and interquartile range of mean relative signal difference across brain regions of interest (ROIs) for [$^{18}$F]FDG and [$^{11}$C]MET subjects, respectively

ROI	[$^{18}$F]FDG	[$^{11}$C]MET
Cerebellum	0.30% (−0.00 to −0.43%)	0.13% (−0.17 to −0.38%)
Frontal cortex	0.11% (−0.09 to −0.49%)	0.07% (−0.07 to −0.32%)
Hippocampus	−0.04% (−0.21 to −0.34%)	0.09% (−0.29 to −0.43%)
Occipital cortex	0.11% (−0.0 to −0.28%)	0.04% (−0.05 to −0.19%)
Parietal cortex	0.19% (0.02 to −0.38%)	0.07% (−0.02 to −0.24%)
Striatum	0.03% (−0.15 to −0.28%)	0.04% (−0.15 to −0.24%)
Temporal cortex	0.13% (−0.01 to −0.26%)	0.05% (−0.11 to −0.19%)
Thalamus	−0.02% (−0.12 to −0.17%)	−0.01% (−0.28 to −0.19%)
Gray matter	0.07% (−0.00 to −0.31%)	0.07% (−0.03 to −0.19%)
White matter	−0.26% (−0.47 to −0.02%)	−0.15% (−0.44 to −0.11%)
Whole brain	−0.00% (−0.09 to −0.15%)	0.02% (−0.05 to −0.08%)

**Fig. 1:**
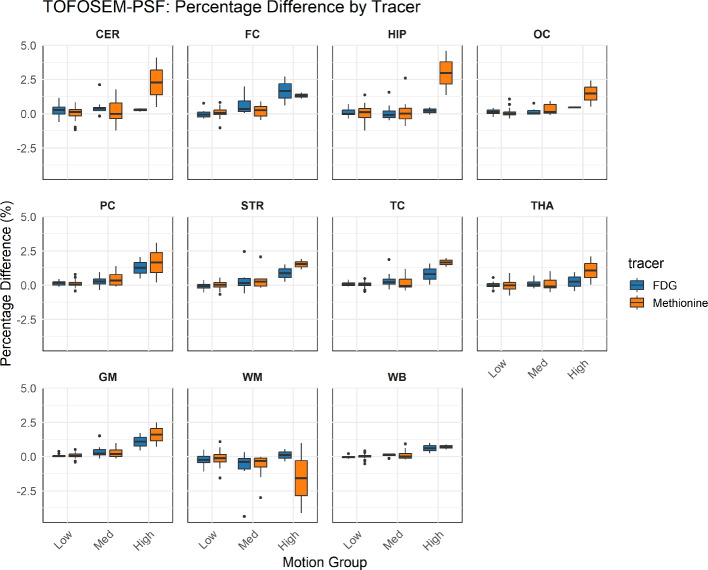
The relative signal difference presented as boxplots for each region of interest (ROI), divided by motion group and radiotracers. ROIs included the whole brain (WB), cerebellum (CER), frontal cortex (FC), hippocampus (HIP), occipital cortex (OC), parietal cortex (PC), striatum (STR), temporal cortex (TC), thalamus (THA), gray matter (GM), and white matter (WM)

Figure 1 presents the relative signal difference for each ROI, divided by motion groups and radiotracers, illustrating how the effect of ddMC varies with motion magnitude as hypothesized. In general, the highest signal difference between noMC and ddMC was found for high motion subjects, followed by medium and low motion subjects. For [$^{18}$F]FDG in the whole brain (WB), the median (interquartile range) was −0.04% (−0.09−0.00%) for low motion, 0.14% (0.06−0.16%) for medium motion, and 0.61% (0.41−0.80%) for high motion. For [$^{11}$C]MET, the median was 0.02% (−0.05−0.07%) for low motion, 0.00% (−0.13−0.21%) for medium motion, and 0.70% (0.61−0.79%) for high motion. Representative images for the high motion category, showing reconstructions with and without MoCo as well as the corresponding difference images, are provided in Supplementary A for both [$^{18}$F]FDG and [$^{11}$C]MET. Evaluating the hypothesis that ddMC performance would be similar across [$^{11}$C]MET and [$^{18}$F]FDG tracers, the Mann–Whitney U tests revealed no statistically significant differences between radiotracers across the various ROIs. As a result, no significant motion-related differences between radiotracers could be identified within ROIs.

Table 2 presents the outcome of the motion estimation and correction by calculating the median normalized cross-correlation ($\overline{XC}$) across low, medium, and high categories, for each tracer distributor, both with and without MC to further evaluate the hypothesis that the effect of ddMC varied with motion magnitude. A value of 0 indicates no correlation between the volumes over time and the reference, while a value of 1 indicates perfect correlation. For all categories and tracer distributors, reconstruction with ddMC resulted in equal or higher median cross-correlation, with greater improvements observed in higher motion categories. The $\overline{XC}$ was higher for [$^{18}$F]FDG compared to [$^{11}$C]MET.

**Table 2:** The median normalized cross correlation ($\overline{XC}$) for the 30 s OSEM-PSF PET frame reconstructions. Four subject were included in each motion category, with equal distribution between radiotracers

	Low motion		Medium motion		High motion	
	[$^{18}$F]FDG	[$^{11}$C]MET	[$^{18}$F]FDG	[$^{11}$C]MET	[$^{18}$F]DG	[$^{11}$C]MET
Without MC	0.98	0.89	0.97	0.82	0.97	0.80
With MC	0.98	0.90	0.98	0.82	0.98	0.85

**Fig. 2:**
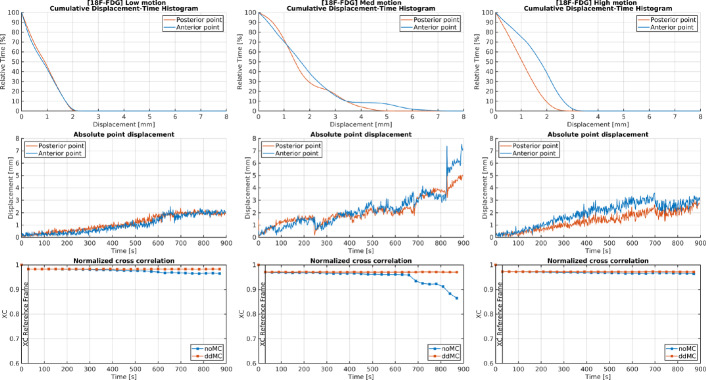
Cumulative displacement-time histograms (cDTH), motion plots generated by lmDuetto, and normalized cross-correlation (XC) curves for 30-second frame reconstructions of one [$^{18}$F]FDG PET subject from each motion category (low, medium, and high). The cDTH and motion plots show the displacement of two points defined at 70 mm anterior and 70 mm posterior to the brain center [19]

**Fig. 3:**
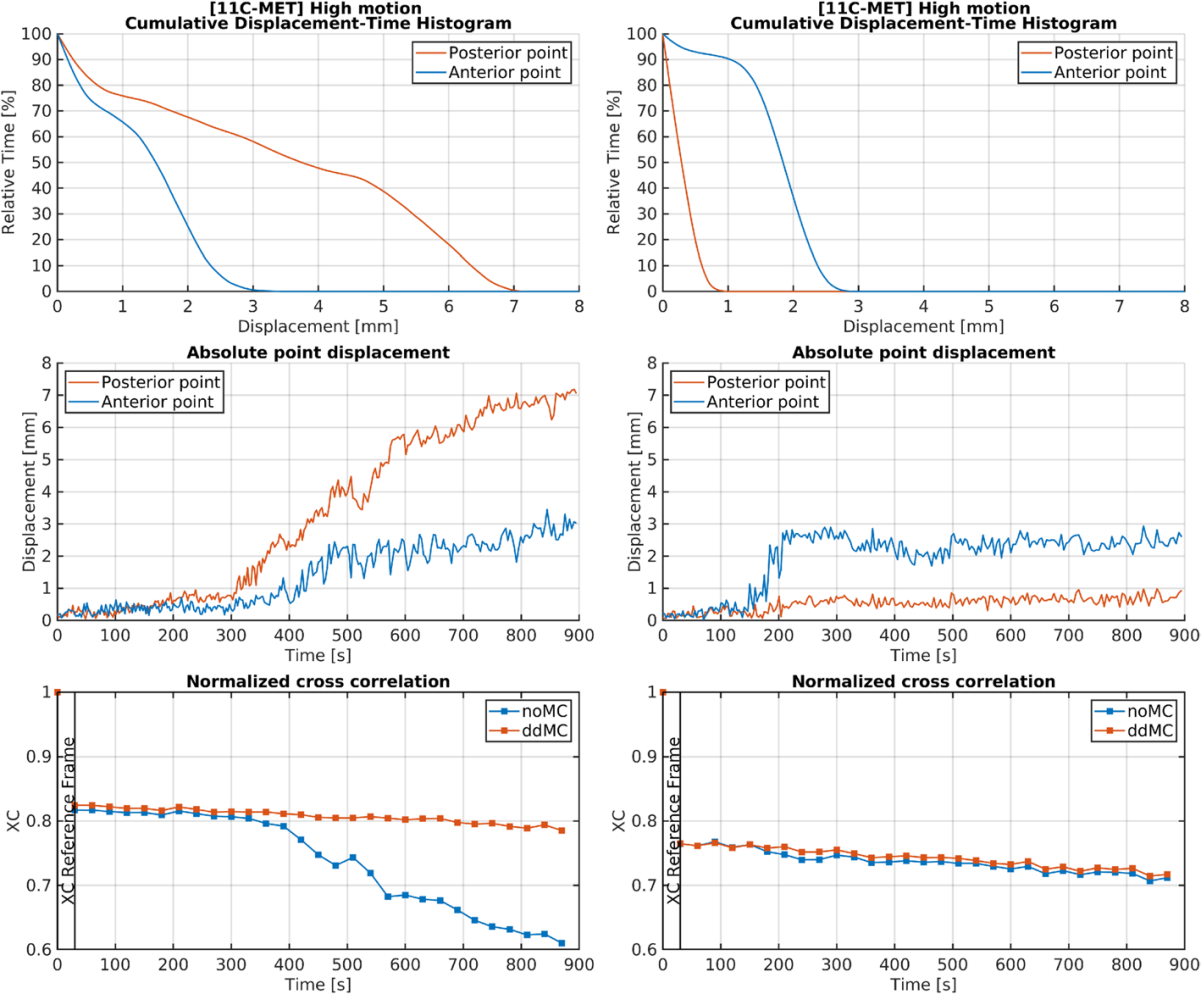
Cumulative displacement-time histograms (cDTH), motion plots generated by lmDuetto, and normalized cross-correlation (XC) for the 30-second reconstructions of high-motion [$^{11}$C]MET PET subjects. The cDTH and motion plots show the displacement of two points defined at 70 mm anterior and 70 mm posterior to the brain center [19]

To evaluate the hypothesis that automatic motion categorization would reflect motion levels impacting PET image quality, Fig. 2 presents the outcome of the motion estimation and automatic motion categorization provided by the lmDuetto software. Assessments were performed using cDTH, motion plots generated by lmDuetto, and XC for the 30-second reconstructions of the one low, one medium, and one high motion categorized [$^{18}$F]FDG PET subject. Figure 3 presents the same analyses (cDTH, motion plots, and XC) for the high motion categorized [$^{11}$C]MET PET subjects. Results for all subjects are provided in Supplementary A. The cDTH enables clearer visualization and simplifies the analysis of motion estimation compared to the motion plots generated by lmDuetto, as it shows the distribution of motion magnitudes over time, allowing to assessment of the extent and frequency of motion during the PET acqusition. A stable XC value over time suggests that the similarity of each 30 s reconstruction aligns with the reference frame. A decreasing XC-value for noMC within frames that temporally correlates with induced motion indicates accurate motion estimation. Conversely, an increase in XC for ddMC, temporally corresponding with increasing motion estimates, suggests successful motion correction. According to the automatic motion classification, motion correction is expected to yield greater increases in XC over time for high motion subjects compared to low motion subjects.

## Discussion

Patient motion remains a key challenge in PET imaging due to relatively long acquisition times. Depending on the patient’s condition, remaining still throughout the scan can be difficult, leading to motion-induced image degradation. This affects both visual quality and the accuracy of quantitative measures. As a result, effective motion correction is essential to ensure reliable image interpretation and analysis. This study has analyzed the quantitative impact of motion correction across multiple brain regions of the commonly used [$^{18}$F]FDG PET tracer and the less commonly used lower-accumulating [$^{11}$C]MET PET tracer. In addition to comparing radiotracers, the analysis evaluated region-specific effects, the impact of motion magnitude, and the accuracy of automatic motion categorization in reflecting motion levels that impact PET image quality. The hypotheses were that ddMC performance would be similar across tracers, that frontal cortical regions would be most affected, that ddMC effects would vary with motion magnitude, and that the commercially proposed automatic motion categorization would reflect motion-related impacts on PET image quality.

Motion correction appeared effective for both [$^{11}$C]MET and [$^{18}$F]FDG PET. While no statistically significant difference was detected, limited data prevent ruling out modest differences in performance. A statistical analysis on motion group level would be preferable, prohibited within this study by the limited number of subjects classified in the high motion category. The introduction of motion correction resulted in overall positive median relative signal differences regardless of radiotracer used (Table 1), consistent with the positive signal changes reported in other studies [14, 19]. The implementation of motion correction slightly increased signal values within cortical ROIs, while subcortical ROIs showed negligible changes. Because brain motion is not purely translational and the center of rotation is typically located posteriorly rather than central, frontal cortical regions were expected to be more affected by motion than other regions. The global white matter ROI had an expected negative median relative signal differences, due to the reduction of dynamic signal mixing caused by motion.

In previous studies, the subject majority was categorized as medium motion [19, 21]. The larger number of subjects categorized as low motion in this study is probably due to the PET/MR acquisition where the subject is immobilized within the head coil, using head phones and cushions, as also reported in the PET/MR study by Neri et al. [14]. Analyzing data with similar temporal resolution from a PET/CT, a more substantial motion might be observed as the subjects are not as fixated.

The impact of ddMC for low and medium motion category is redundant, both according to median relative signal difference (Figure 1) and selected subject’s XC evaluation (Table 2). In the high motion category, applying ddMC resulted in median relative signal differences within a clinically relevant range for certain ROIs (Figure 1), and an improvement in XC (Table 2). This indicates that motion correction for this category is a necessity. As the majority of evaluated subjects (96% corresponding to category low or medium motion) did not have clinically relevant mean relative signal difference, a pipeline with automatic offline ddMC reconstructions of approximately 3 h (depending of hardware available) is undesired. A pipeline of motion estimation could however be implemented to indicate if full ddMC reconstruction is beneficial. This requires a sophisticated reporting of the motion estimation.

To estimate the accuracy of the ddMC, XC values over time is usable tool as a correlation between increased XC and motion within the motion plots indicates accurate motion estimation by lmDuetto (seen in Figs. 2 and 3). A drawback is the necessity of both noMC and ddMC reconstructions and the dynamic 30 s reconstruction, which is time consuming (approx 9 h). A potential strategy to save time and enhance the efficiency of the XC evaluation would be to identify substantial motion within the motion estimation and depending on the motion, adapt the time length of the dynamic reconstruction.

Figure 2 presents cDTH, motion plots generated by lmDuetto, and XC for the 30-second reconstructions of selected [$^{18}$F]FDG subjects categorized by motion levels. According to the automatic motion classification, the effect of motion correction was expected to increase from left to right. However, looking at the XC-evaluation, subjects classified as having low and high motion were the least affected in XC applying ddMC, whereas the subject with medium motion showed the most improvement. The impact of motion correction also varied among [$^{11}$C]MET subjects classified as high motion (Figure 3). Although the subjects were categorized as high motion, ddMC increased the XC value over time for only one of them. Since lmDuetto classifies motion based on the median absolute displacement, it will be non-observant for severe but short-duration motion periods occurring during less than half of the PET acquisition time, misclassifying the subject. To address this, we suggest to additionally present cDTH to easily visualize the impact of ddMC compared to noMC (Figs. 2 and 3). For example, looking at the absolute point displacement for the second [$^{11}$C]MET high motion subject, there was an early rapid one time movement (the step gradient from 0.3mm to 2.8mm of the anterior point in Figure 3), hence the impact of the motion was not substantial, yet classified as high motion as the absolut median >2 mm. The corresponding cDTH illustrates that all posterior time points and 90 % of the anterior time points had a <1 mm displacement, easily estimating that ddMC for this subject would have low impact, which the indifference in XC values between noMC and ddMC confirmed. The other [$^{11}$C]MET high motion subject was drifting gradually, where the ddMC is highly important according to the XC-evaluation. For this subject, the cDTH shows that only 50% of the anterior time points were <1.5mm and the posterior time points <3.5mm, indicating substantial motion influence on the reconstructed data. The presentation of cDTH for high motion is based on the motion estimation and is simply a redistribution of the information already obtained from lmDuetto, making it easily implemented.

A limitation of the study was the potential inaccuracy in MRI-to-PET registration, which could impact the evaluation of motion correction within specific ROIs. To mitigate this, visual assessment was performed, and initial alignment were applied to enhance registration robustness. The analysis confirmed that all registrations resulted in displacements $\le$1 mm. An additional limitation was the limited number of subjects classified in the high motion category, potentially reducing the sensitivity for detecting motion-related effects in this subgroup. Additionally, dividing PET acquisitions into shorter time frames enables inter-frame motion correction, which can reduce motion-induced image degradation compared to a single static reconstruction. The added benefit of ddMC over such an approach would be reduced for subjects with infrequent, discrete movements occurring between frames. However, inter-frame correction cannot account for intra-frame motion, which can still be substantial within a 5-minute window. The benefit of ddMC lies in its ability to correct motion at a finer temporal scale, making it most valuable for subjects with frequent or continuous motion.

In conclusion, our results confirm previous findings with the investigated motion correction strategy for both [$^{18}$F]FDG and [$^{11}$C]MET, and demonstrate that this strategy is suitable for lower accumulating radiotracers such as [$^{11}$C]MET. The quantitative analysis indicated a minor impact of motion correction for subjects categorized as low and medium motion, with greater impact for high motion classified subjects. Regional analysis suggested that cortical regions were slightly more affected by motion than subcortical regions. Finally, the commercially proposed automatic motion categorization of subjects may need re-evaluation (i.e. cumulative displacement-time histograms), as the motion classification does not necessarily correlate with motion levels that substantially affect the PET image quality.

## Supplementary information:

This article includes supplementary material containing additional results.

## Additional file

Supplementary file 1 (pdf 4354 KB)

## Data Availability

The datasets generated during and/or analyzed during the current study are available from the corresponding author on reasonable request.

## Abbreviations

BSREM: Block sequential regularized expectation maximization
CER: Cerebellum
cDTH: Cumulative displacement-time histogram
COD: Centroid-of-distribution
ddMC: Data-driven motion correction
FC: Frontal cortex
FDG: [$$^{18}$$F]Fluorodeoxyglucose
GM: Gray matter
HIP: Hippocampus
MET: [$$^{11}$$C]methionine
MRI: Magnetic resonance imaging
noMC: Without motion correction
OC: Occipital cortex
OSEM-PSF: Ordered subsets expectation maximization with point spread function correction
PC: Parietal cortex
PET: Positron emission tomography
ROI: Region of interest
STR: Striatum
TC: Temporal cortex
THA: Thalamus
WB: Whole brain
WM: White matter
XC: Normalized cross correlation
ZTE: Zero echo time
