# Supplementary material for: The impact of data driven motion correction for clinical brain PET/MRI radiotracers
Source: EJNMMI Phys. 2026 Apr 15;13:35. doi: 10.1186/s40658-026-00863-7 (PMC13083739; doi:10.1186/s40658-026-00863-7)
Supplement: Supplementary file 1 — (pdf 4354 KB) [file 40658_2026_863_MOESM1_ESM.pdf]

Supplementary information

A. Additional results

Figure A.1 shows representative axial, coronal, and, sagittal images from the high motion category for both [^{18}F]Fluorodeoxyglucose (FDG) and [^{11}C]methionine (MET). Images reconstructed without and with motion correction (MoCo) are displayed alongside the corresponding relative difference images, illustrating the spatial distribution and magnitude of changes introduced by motion correction in subjects with estimated head movement categorized as high by LmDuetto.

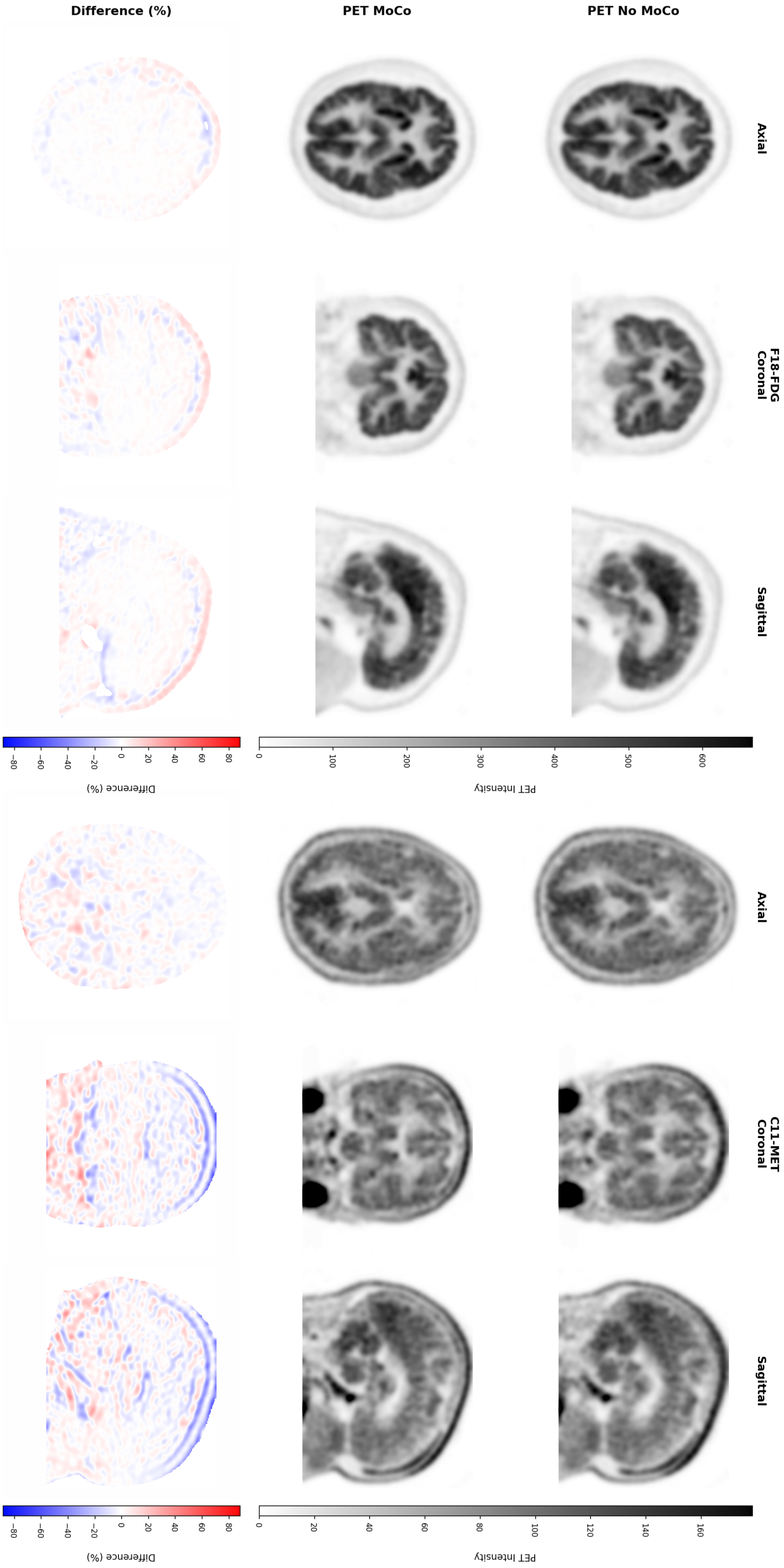

Figure A.1: Representative axial, coronal, and, sagittal images from the high motion category for [^{18}F]Fluorodeoxyglucose (FDG) (left) and [^{11}C]methionine (MET) (right). For each tracer, images reconstructed without motion correction (PET No MoCo), with motion correction (PET MoCo), and the corresponding difference images (PET MoCo − PET No MoCo) are shown.

Figure A.2 and A.3 present the normalized cross-correlation (XC) over time and motion plots automatically generated by lmDuetto, along with cumulative displacement-time histograms (cDTH), for the 30-second reconstructions of all twelve subjects categorized into low, medium, and high motion groups for [^{18}F]FDG and [^{11}C]MET radiotracers, respectively. An increase in XC for ddMC, aligned with the corresponding increasing motion estimates in the motion plots, indicates successful motion correction. The cDTH provide clearer visualization and simplify the analysis of the impact of motion estimation on PET data.

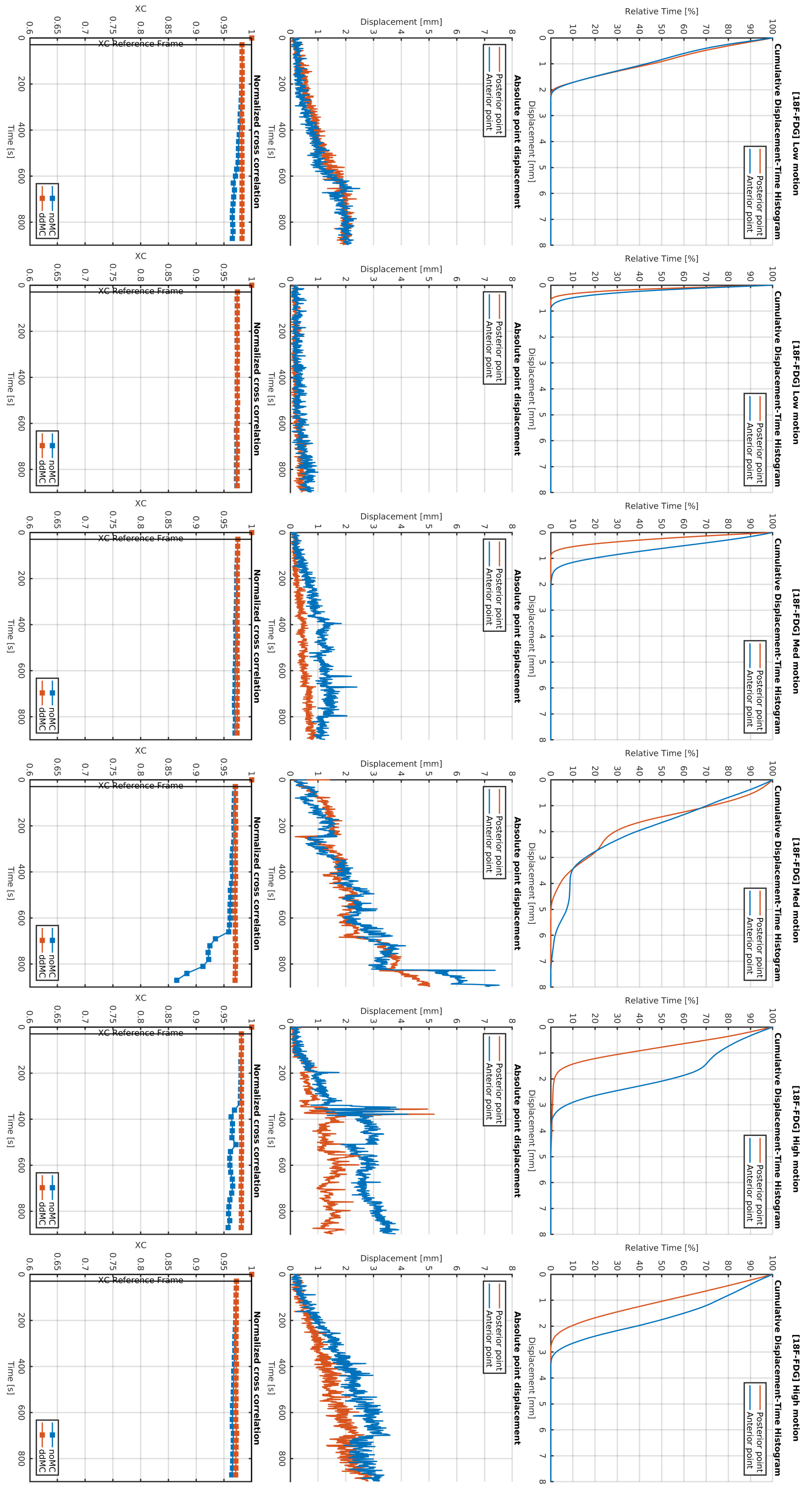

Figure A.2: Cumulative displacement-time histograms (cDTH), motion plots generated by lmDuetto, and normalized cross-correlation (XC) for the 30-second reconstructions of all twelve high-motion [^{18}F]Fluorodeoxyglucose (FDG) subjects categorized as low, medium, and high motion groups.

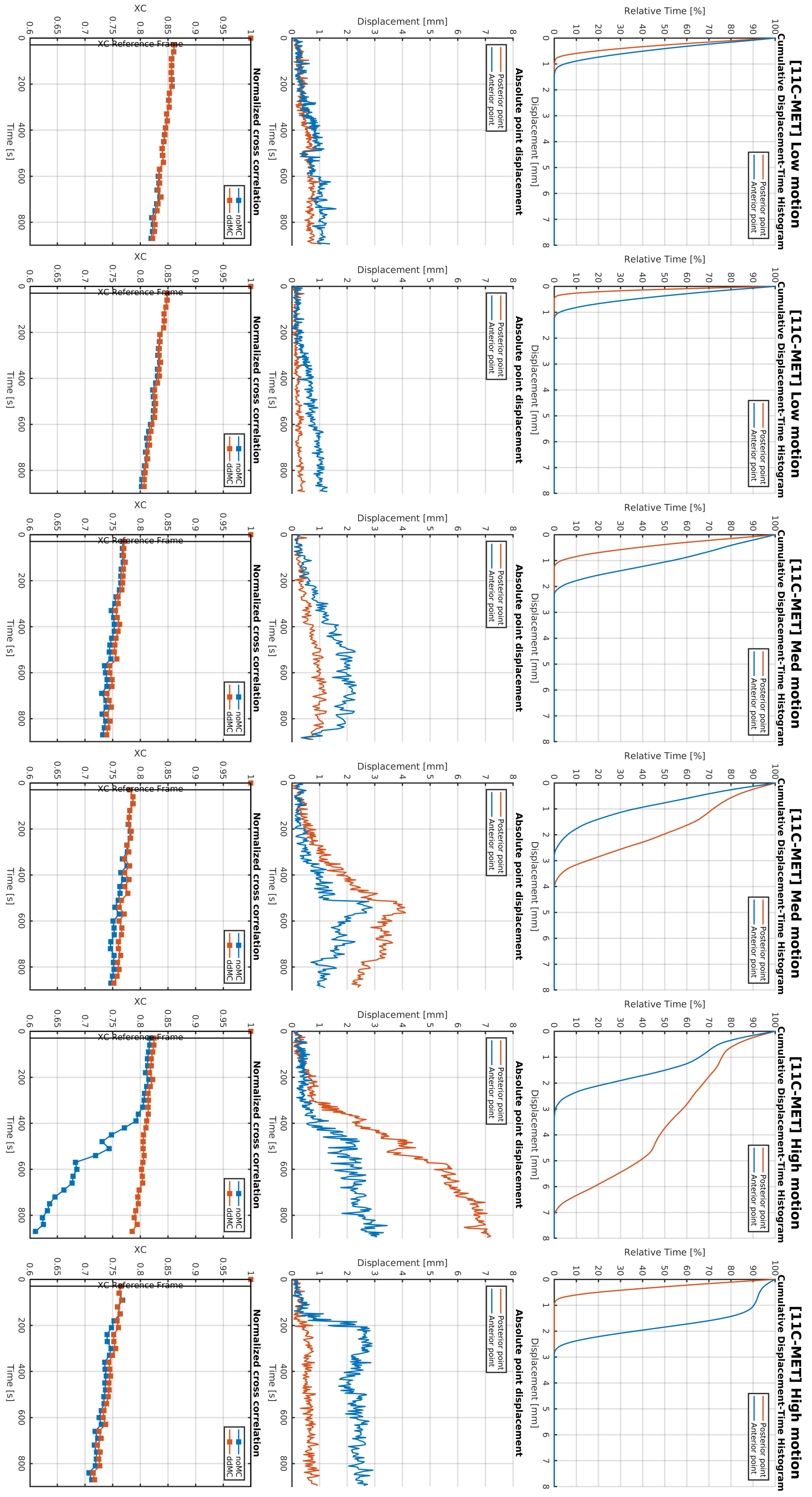

Figure A.3: Cumulative displacement-time histograms (cDTH), motion plots generated by lmDuetto, and normalized cross-correlation (XC) for the 30-second reconstructions of all twelve high-motion [^{11}C]methionine (MET) subjects categorized as low, medium, and high motion groups.
